# Serum From Preeclamptic Women Triggers Endoplasmic Reticulum Stress Pathway and Expression of Angiogenic Factors in Trophoblast Cells

**DOI:** 10.3389/fphys.2021.799653

**Published:** 2022-02-04

**Authors:** Karla R. Castro, Karen M. Prado, Aline R. Lorenzon, Mara S. Hoshida, Eliane A. Alves, Rossana P. V. Francisco, Marcelo Zugaib, Aldilane L. X. Marques, Elaine C. O. Silva, Eduardo J. S. Fonseca, Alexandre U. Borbely, Mariana M. Veras, Estela Bevilacqua

**Affiliations:** ^1^Laboratory for Studies in Maternal-Fetal Interactions and Placenta, Department of Cell and Developmental Biology, Institute of Biomedical Sciences, University of São Paulo, São Paulo, Brazil; ^2^Huntington Medicina Reprodutiva—Eugin Group, São Paulo, Brazil; ^3^Department of Obstetrics and Gynecology, School of Medicine, University of São Paulo – HCFMUSP, São Paulo, Brazil; ^4^Cell Biology Laboratory, Institute of Health and Biological Sciences, Federal University of Alagoas, Maceio, Brazil; ^5^Optics and Nanoscopy Group, Institute of Physics, Federal University of Alagoas, Maceio, Brazil; ^6^Laboratory of Experimental Air Pollution, Department of Pathology, School of Medicine, University of São Paulo, São Paulo, Brazil

**Keywords:** ER stress, UPR pathway, trophoblast cells, pré-eclampsia, angiogenic factors

## Abstract

Preeclampsia (PE) is a hypertensive disease of pregnancy-associated with placental cell death and endoplasmic reticulum (ER) stress. It is unknown whether systemic factors aggravate placental dysfunction. We investigated whether serum factors in pregnant women with PE activate ER stress and unfolded protein responses (UPRs) in placental explants and trophoblast cells lineage. We cultured placental explants from third-trimester term placentas from control non-preeclamptic (NPE) pregnant women with serum from women with PE or controls (NPE). In PE-treated explants, there was a significant increase in gene expression of *GADD34*, *CHOP*, and *SDF2*. At the protein level, GRP78, SDF2, p-eIF2α, and p-eIF2α/eIF2α ratio were also augmented in treated explants. Assays were also performed in HTR8/SV-neo trophoblast cell line to characterize the putative participation of trophoblast cells. In PE serum-treated protein levels of p-eIF2a and the ratio p-elF2 α/elF2α increased after 12 h of treatment, while the gene expression of *GADD34*, *ATF4*, and *CHOP* was greater than control. Increased expression of SDF2 was also detected after 24 h-cultured HTR8/SV-neo cells. PE serum increased *sFLT1* gene expression and decreased *PlGF* gene expression in placental explants. Morphologically, PE serum increased the number of syncytial knots and reduced placental cell metabolism and viability. Analysis of the serum of pregnant women with PE through Raman spectroscopy showed changes in amino acids, carotenoids, lipids, and DNA/RNA, which may be associated with the induction of ER stress found in chorionic villi treated with this serum. In conclusion, this study provides evidence that the serum of pregnant women with PE may impact placental villi changing its morphology, viability, and secreted functional factors while triggers ER stress and an UPR. The differences between PE and control sera include molecules acting as inducing factors in these processes. In summary, the results obtained in our assays suggest that after the development of PE, the serum profile of pregnant women may be an additional factor that feeds a continuous imbalance of placental homeostasis. In addition, this study may expand the possibilities for understanding the pathogenesis of this disorder.

## Introduction

Preeclampsia (PE) is a significant cause of maternal and perinatal morbidity and mortality, affecting 5–8% of pregnant women worldwide (Svein et al., 2017). It is characterized by arterial hypertension and may be accompanied by proteinuria and kidney, liver, or placenta failure (Brown et al., 2018). Symptoms appear from the 20th week of gestation (usually associated with the most severe disease cases) and usually disappear after birth with complete removal of the placenta. PE is associated with systemic activation of endothelial cells (endothelial dysfunction, vasoconstriction, and increased vascular permeability) followed by hypoperfusion of various organs, including the fetal-placental unit (Redman et al., 2020). The most widely accepted theory about this syndrome in severe cases includes defects in spiral artery remodeling during placenta development leading to the maintenance of high-resistance uterine vessels throughout gestation (Roberts, 1998).

Previous investigations showed that angiogenic factors such as placental growth factor (PlGF) and the soluble fms-like tyrosine kinase-1 (sFlt-1) participate in the development of the disease (Stepan et al., 2015). High serum levels of sFlt-1 and low levels of PlGF appear to compromise angiogenic processes and contribute to inadequate trophoblast invasion; the result is hypoperfusion of the placenta and hypoxia at the maternal-fetal interface (Tomimatsu et al., 2019).

Endoplasmic reticulum (ER) stress has been associated with the pathophysiology of severe PE. Human term placentas from intrauterine growth-restricted newborns and pregnant women with severe preeclampsia highly express ER stress markers, suggesting that this pathway may contribute to abnormal placental development and function (Burton et al., 2009; Burton and Yung, 2011). ER stress responses may be triggered by environmental factors such as hypoxia and nutrient deprivation that activate signaling pathways, which together are called the unfolded protein response (UPR) (Walter and Ron, 2011; Metcalf et al., 2020). UPR activation is associated with reduced translation rates, increased expression of chaperones and foldases, and activation of ER-associated degradation (Xu et al., 2005; Scriven et al., 2009). ER stress activates several cellular responses to rescue cellular homeostasis or induce apoptosis of injured cells, preventing the secretion of inactive or corrupted (i.e., non-functional) proteins to the extracellular environment (Xu et al., 2005; Sano and Reed, 2013). Evidence suggests that the interruption of protein homeostasis in the ER results in disordered cellular responses, contributing to the pathogenesis of several diseases (Cao et al., 2016; Yang et al., 2016), including placental pathologies such as intrauterine growth restriction, severe preeclampsia, and gestational diabetes mellitus (Bastida-Ruiz et al., 2017). This response begins with the recruitment of the GRP78/BiP chaperone, which in non-stressing conditions is coupled to three resident’s ER sensor proteins [PKR-like eukaryotic initiation factor 2a kinase (PERK)], inositol-requiring enzyme 1 (IRE1), and activating transcription factor 6 (ATF6) (Yoshida, 2007). Under stress conditions, the decoupling between BiP/sensor proteins consequently activates the UPR pathway. Stromal-derived factor 2 (SDF2) has also been related to the activation of UPR pathways. Previous studies suggested that SDF2 expression is associated with controlling cell survival/apoptosis mediated by the PERK arm (Lorenzon-Ojea et al., 2016). In addition, SDF2 was upregulated in the most severe cases of this disease (Lorenzon-Ojea et al., 2020; Lorenzon et al., 2021).

The pathophysiology of PE and the factors that aggravate a pregnant woman’s inflammatory and general health status have been widely discussed. Serum profiles of growth factors, cytokines, and other regulatory molecules change dramatically during PE, which hints at the significant role these factors may play in various degrees of endothelial dysfunction and consequently in the pathogenesis of this disorder (Lima et al., 2011; Park et al., 2011; Mori et al., 2014). In this context, our hypothesis assumes that factors present in the maternal serum of preeclamptic women activate ER stress pathways in the villous trophoblast, unbalancing the expression of vasoactive products that characterize the preeclamptic environment, perpetuating or worsening placental stress. To address this possibility, we measured the impact of PE serum on ER stress pathways and the expression of angiogenic factors in placental explants of 3rd-trimester chorionic villi and in a non-malignant trophoblast cell line.

## Materials and Methods

### Ethics Approval

The Ethics Committee from the University of São Paulo and the Brazilian National Ethics Committee of Human Experimentation approved the study (CAAE no. 774.907). The villus samples were obtained from term human placentas from women without medical or obstetrical complications who delivered a healthy singleton neonate at term after elective, non-labored, cesarean section, at the Hospital das Clínicas of the University of São Paulo/Brazil. Written informed consent was obtained from all participants before the procedures.

### Patients and Sample Collection

The cohort consisted of 14 pregnant women with prenatal care and delivery at the Hospital das Clinicas of the University of São Paulo. Inclusion criteria for each group were determined as follows: (i) *PE group* (*n* = 7): pregnant women diagnosed clinically with severe preeclampsia [defined as arterial hypertension (≥ 160/90 mmHg) and proteinuria of ≥ 300 mg/24 h]. All symptoms such as proteinuria, hypertension, and others were early found after the 20th-gestation week (gw), and delivery occurred between weeks 24 and 35 (early onset PE). (ii), *NPE group*, controls (*n* = 7): Pregnant women with normal blood pressure, without proteinuria, without diagnosed chronic diseases [that could predispose the pregnant woman to PE (hypertension, renal disease, obesity, or diabetes)], non-smoking, without alcohol or medication of continuous use, with negative serological reactions for HIV, syphilis, and other infections.

Placentas were obtained from elective cesarean sections at term or indicated for pregnant women with PE (PE group). Placental fragments were collected immediately after the C-section in phosphate-buffered saline (PBS; 0.1 M, pH7.4) supplemented with antimicrobials [penicillin (100 IU/mL), streptomycin (100 μg/mL), gentamicin (5 μg/mL), and amphotericin B (25 μg/mL)].

Blood samples were obtained at delivery before elective cesarean section from PE (*n* = 7) and non-preeclamptic (*n* = 7) groups. The samples were centrifuged at 4°C and 2,000 rpm for 30 min to obtain serum maintained at –80°C until use.

### Placental Explant Isolation and Culture

Terminal chorionic villi exclusively from non-preeclamptic patients were isolated from the chorionic tree (floating explant model, Miller et al., 2005), washed in sterile PBS with antibiotics, weighed, and immediately cultured in DMEM-F12 medium supplemented with 10% fetal bovine serum (FBS) and antimicrobials (100 U/mL penicillin, 100 μg/mL streptomycin, 5 μg/mL gentamicin, and 2.5 mg/mL amphotericin B), in an incubator at 37°C, in a humid atmosphere containing 5% CO_2_ for 24 h. Before receiving the dissecting villi, the culture plates were covered with fibronectin (10 μg/mL in PBS). After 12 h, the medium was replaced with DMEM-F12 medium containing 10% maternal serum from the control and PE groups. They remained for an additional 24 h under the same culture conditions described above. Placental explants were collected on 4% paraformaldehyde in PBS for morphological analysis, on ice-cold RIPA buffer for western blot, in RNA later^®^ (Thermo Fisher Scientific, Waltham, Massachusetts, EUA) for RT-qPCR assays, and for cellular viability and metabolism evaluation [3- (4,5-dimethylthiazol-2yl) –2,5-diphenyltetrazolium (MTT) and lactate dehydrogenase (LDH) assays, respectively].

### HTR8/SV-Neo Culture

To determine the participation of trophoblast cells in our results, assays were also performed with HTR8/SV-neo cell line, kindly provided by Charles Graham, Ph.D. (Queen’s University, Kingston, Ontario, Canada). Cells were plated in 24-well dishes (2 × 10^4^ cells/well) and maintained in RPMI medium supplemented with 10% FBS and antimicrobials (gentamicin and amphotericin) at 37°C and 5% CO_2_. The medium was then replaced with a new one containing 10% non-preeclamptic serum (NPE-S) or PE serum (PE-S). The cells remained in culture conditions for 12 and 24 h and were then collected for evaluation. The experiments were performed in triplicate.

### 2,5-Diphenyltetrazolium Test

Cellular mitochondrial metabolism of placental explants treated with maternal sera was determined using the MTT test. Explants were cultured as described above. After a 24-h incubation period, the medium was replaced by 30 μL of MTT solution in 300 μL of RPMI without phenol and incubated at 37°C for another 2 h. The medium was then carefully aspirated, and 200 μL of dimethyl sulfoxide was added to each well on a shaker to lyse the cells and solubilize the formazan crystals. Absorbance was determined spectrophotometrically at 570 nm using a reference wavelength of 630 nm on a SpectraMax Plus 384 microplate reader (Molecular Devices). For each placenta obtained [*n* (7)], assays were performed in triplicate. Data were expressed as mean (standard deviation).

### Lactate Dehydrogenase Assay

This assay was carried out using the Pierce LDH Cytotoxicity Assay kit (Pierce) according to the manufacturer’s instructions. HTR8/SV-neo cells [*n*(6)] and chorionic explants [*n*(7)] were plated in triplicate and treated with the serum of NPE or PE pregnant women for 24 h. The conditioned media from experiments and controls in triplicate were transferred to 96-well flat-bottom plates. Additional negative controls were prepared as follows: (i) only complete culture medium; (ii) medium without serum; and (iii) supernatants of explants previously treated with sterile ultra-pure water (50 μL, 45 min, 37°C). As a positive control, explants were treated with lysis buffer (50 μL, 1:10 v/v). All samples received 100 μL of Reaction Mixture (10 min, at 37°C), then 50 μL of Stop Solution. The reading was performed on the SpectraMax Plus 384 Absorbance Microplate Reader (Molecular Devices) at 490 and 690 nm. Cytotoxicity was calculated as follows:


(1) $$\%Cytotoxicity=\frac{O\,D\,s\,a\,m\,p\,l\,e\,s-O\,D\,n\,e\,g\,a\,t\,i\,v\,e\,c\,o\,n\,t\,r\,o\,l}{O\,D\,p\,o\,s\,i\,t\,i\,v\,e\,c\,o\,n\,t\,r\,o\,l-O\,D\,n\,e\,g\,a\,t\,i\,v\,e\,c\,o\,n\,t\,r\,o\,l}\times 100$$

### Morphological Analysis

Cultured explants were fixed in 70% methanol, 20% chloroform, and 10% acetic acid, following the routine procedures for inclusion in Histosec (Merck, Darmstadt, Germany). Five-micron sections adhered to glass slides treated with a 1% silane solution (3-aminopropyl trimethoxysilane, Sigma Chemical Co., St. Louis, MO, United States). The sections were then subjected to morphology characterization and syncytial knot counting using the hematoxylin-eosin staining technique under light microscopy (Axioskop, Zeiss, Germany). Syncytial knots were measured in ten random quadrants of each histological section at 200 × magnification. Samples from three placentas were analyzed per experimental and control group carried out at different times. The values obtained in each experiment were expressed as mean syncytial knots/chorionic villi.

### Quantitative Reverse Transcription-Polymerase Chain Reaction

A PureLink™ RNA Mini Kit (Invitrogen) was used to extract total RNA from placental explants (24-h-cultured, *n* = 7) and HTR8/SV-neo cells (2, 6, 12, and 24-h-cultured, *n* = 6) according to the manufacturer’s instructions. RNA concentration was determined using the NanoDrop 1000 system (Thermo Fisher Scientific) at 230, 260, and 280 nm. Total RNA (1 μg) was used for reverse transcription using a High-Capacity RNA-to-cDNA kit (Applied Biosystems, Foster City, CA, United States) following the manufacturer’s instructions. For quantitative RT-PCR (qPCR), PowerUP SYBR Green PCR Master Mix™ (Applied Biosystems^®^, Invitrogen) was used with appropriate primer pairs (Supplementary Table 1) using a Step One Plus™ thermocycler (Applied Biosystems, Foster City, CA), according to the manufacturer’s protocols.

Thermo-cycling parameters for PCR amplification were as follows: 10 min at 95°C followed by 40 cycles of 15 min at 95°C, 1 min at 60°C, and 30 s at 95°C. The specificity of the amplification was determined by melting curve analysis (55–95°C, held and read every 10 s at each 0.5°C increment). Primers for *PlGF, sFlt-1, SDF2, sXBP1, ATF4, CHOP*, and *GADD34* mRNAs are listed in Supplementary Table 1. Expression of mRNA was calculated according to the 2–ΔΔCt method, normalized to the *YWHAZ* gene and expressed as fold-change relative to control.

### Western Blot Analysis

Total protein of HTR8/SV-neo cells and placental explants cultured with PE and NPE serum were homogenized (Precellys^®^ tissue homogenizer) in ice-cold RIPA buffer (1% NP-40, 0.25% Na-deoxycholate, 150 mM NaCl, 1 mM EDTA, 1 mM PMSF, 50 mM Tris-HCl, pH 7.4) supplemented with protease and phosphatase inhibitor cocktail (Sigma). The lysates were centrifuged for 15 min at 12,000 rpm, 4°C, and the supernatants were collected and subjected to western blot analysis. According to the manufacturer’s instructions, protein concentrations were quantified using the Pierce BCA Protein Assay Kit (Thermo Fisher Scientific, Rockford, IL). Samples were separated by 10% sodium dodecyl sulfate-polyacrylamide gel electrophoresis and transferred to nitrocellulose membranes (0.45 mm; Bio-Rad). Membranes were blocked in 3% non-fat milk (Bio-Rad) for 1 h and incubated overnight with primary antibodies against GRP78, eIF2α, p-eIF2α, and SDF2 (Supplementary Table 2). Antibodies were diluted in tris-buffered saline (TBS) 3% non-fat milk powder. The samples were subsequently incubated with an antibody to β-actin as a loading control. The membranes were washed in TBS-Tween 20 and incubated for 1 h with secondary antibodies (Supplementary Table 2). Signals were detected using a chemiluminescent solution (Clarity™ ECL Western Blotting Substrate Bio-Rad) as instructed by the manufacturer and developed using a G: Box Chemi HR (Syngene). The bands were quantified by densitometry using Imagej^®^ software 1.43 (NIMH, NHI, Bethesda, MD, United States). Levels of protein were normalized to values for β-actin.

### Analysis of Preeclampsia Serum Using Raman Spectroscopy

The Raman spectra were measured using an optical microscope (BXFM, Olympus, Japan) coupled to an XploRA spectrometer (Horiba, Japan) and equipped with a 532-nm laser that was focused on cell nuclei through a 100 × objective (NA = 0.9). The same objective lens was used for collecting Raman scattered light after interaction with the sample in backscattering geometry. The frequency calibration was set by reference to the 520 cm^–1^ vibrational band of a silicon wafer. Under the same conditions, five Raman spectra were captured from each sample of both groups in the spectral range of 600–1,800 cm^–1^. To minimize laser-induced heating of the specimens, low-power irradiation of 5 mW at the sample surface was used during a short exposure time (3-s laser exposure for five accumulations). The diffraction grating had 1,200 lines/mm, which yielded a spectral resolution of 1.5 cm cm^–1^. All spectra were smoothed, background-adjusted, and normalized using an algorithm implemented in MatLab software (Mathworks, Natick, MA, United States) for data preprocessing and spectral analyses. Therefore, the external noises were suppressed, and the useful information about the biochemical composition was enhanced. The fluorescence background was removed from the spectra, and principal component analysis (PCA) was performed to evaluate the spectral variability in the dataset. All multivariate statistical analyses were implemented using MatLab software. Raman individual bands with statistically significant fluctuations were isolated for further comparison.

### Statistical Analysis

All experiments were repeated at least three times independently. Data were expressed as means ± SD. Each data set was tested for normal distribution using the Kolmogorov-Smirnov test and for homogeneity of variances before statistical analysis. Statistical analyses were performed using GraphPad Prism 5.0 software (GraphPad Software, San Diego, CA, United States). Data were analyzed using the unpaired Student’s *t*-test comparing PE-treated groups with controls and one-way analysis of variance followed by Tukey or Mann-Whitney *post hoc* test for comparisons of multiple groups. Differences of *p* < 0.05 were considered significant. MatLab software was used for Raman spectroscopy for spectral analysis, and PCA was used for multivariate spectral analyses. The primary altered spectra were also analyzed using the two-tailed Mann-Whitney test (GraphPad Prism). Data were represented as means ± SEM. The minimal level of significance for all experiments was set at *p* < 0.05.

## Results

### Characteristics of the Study Population

Clinical characteristics and gestational outcomes, including maternal age, gestational age, number of pregnancies, number of previous deliveries, placental weight, blood pressure (systolic and diastolic), proteinuria, and birth weight neonates, are shown in Table 1. Although the maternal age was similar between the groups, there were significantly higher values for gestational age, the number of pregnancies, and the number of previous deliveries in NPE patients than patients with PE (*p* < 0.05). Blood pressure levels in patients with PE were higher than in NPE patients (*p* < 0.005, systolic and *p* < 0.05, diastolic). Proteinuria was evaluated only in pregnant women with PE and presented an average of 5.2 g/L in 24 h. We also found significant differences among placental and fetal weight in the PE group (*p* < 0.005). All patients with PE used antihypertensive agents such as methyldopa, amlodipine, carvedilol, and hydralazine.

**TABLE 1:** Clinical characteristics of controls and patients with pre-eclampsia (PE).

	NPE (*n* =7)	PE (*n* =7)
^#1^Maternal age	29.14 ± 1.62	26.43 ± 2.48
^#1^Gestational week	39 ± 0.30	34.04 ± 1.27*
^#2^Number of pregnancies	3.28 ± 0.40	1.28 ± 0.18*
^#2^Previous deliveries	1.85 ± 0.60	0.28 ± 0.18*
^#1^Placental weight (g)	493.7 ± 52.18	255.3 ± 36.66**
^#1^Proteinuria (24 h g/L)	NA	5.2
^#2^Blood pressure (mmHg)		
Systolic	117.5 ± 4.78	180.3 ± 10.22**
Diastólic	72.5 ± 2.5	107 ± 5.13*
^#2^Birth weight (kg)	3.32 ± 0.13	2.2 ± 0.19**

*Data presented as the mean ± standard deviation.*

*^#1^Student’s t-test.*

*^#2^ Mann–Whitney U-Test.*

*/** Significantly different in relation to the data found for non-preeclamptic (Non-PE) pregnant women; (PE) preeclampsia. *p < 0.05; **p < 0.005.

### Preeclampsia Serum Induced Gene and Protein Expression of Endoplasmic Reticulum Stress and Unfolded Protein Response Biomarkers in Placental Cells

Induction of ER stress by PE serum was evaluated in explants of chorionic villi and the trophoblast cell line HTR8/SV-neo by investigating the mRNA of ER stress and UPR targets and downstream genes and proteins, as well as the phosphorylation of eIF2α and the splicing of sXBP1.

**FIGURE 1:**
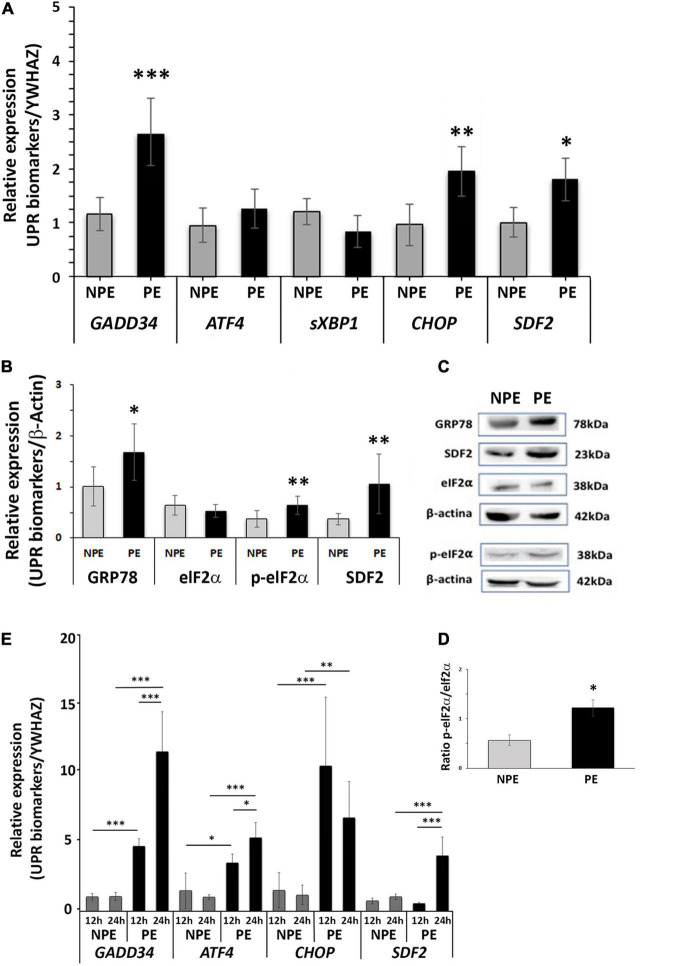
Gene expression **(A)** and protein **(B–D)** analysis of placental explants (24-h-cultures) and gene expression of HTR8/SV-neo cells (**E:** 12 and 24-h-cultures) cultured with PE serum (PE, black bars) and with serum from non-preeclamptic pregnant women (NPE, white bars). **(A)** *GADD34*, *CHOP*, and *SDF2* showed higher levels in comparison to respective controls. **(B)** The explants of PE serum-treated showed higher expression of *GRP78*, p-eIF2α, and *SDF2* than the respective controls. The values were normalized to β-actin. **(C)** GRP78, SDF2, eIF2α, p-eIF2a, and β-actin representative blots from placental explants. **(D)** Increased ratio p-eIF2 alpha: Total eIF2 alpha is seen in samples from PE-treated explants. **(E)** PE serum increased the gene expression of GADD34, CHOP, and SDF2 in HTR8/SV-neo cells. The data are represented as mean ± SD (*n* = 7). Data from **(A–D)** were analyzed using the unpaired Student’s *t*-test followed by the Mann-Whitney posttest. Times of the HTR8/SV-neo cultures were analyzed using analysis of variance and Tukey’s multiple comparisons *post hoc* test. ^∗^*p* < 0.05, ^∗∗^*p* < 0.005, ^∗∗∗^*p* < 0.0005.

As shown in Figure 1A (and Supplementary Table 3), there was an increase in mRNA of the UPR target genes *GADD34* (*p* < 0.0001), *CHOP* (*p* = 0.0015), and *SDF2* (*p* = 0.0256), whereas there was no response to PE serum for *ATF4* and *sXBP1* after 24 h of serum treatment. In line with the effect of PE serum on UPR target gene induction, relative protein levels (Figures 1B,C) of GRP78 (*p* < 0.0474) and p-eIF2α (*p* < 0.0039) were induced by 24 h treatment in placental explants (as was the ratio p-eIF2α/eIF2α, *p* < 0.0001, Figure 1D), whereas the protein level of total elF-2α was unchanged (Figures 1B,C and Supplementary Table 3). These results suggest the onset of ER stress (Walter and Ron, 2011; Lorenzon-Ojea et al., 2020; Metcalf et al., 2020).

To specifically assess the participation of trophoblast cells in the villous ER stress activation, we used the trophoblast cell line HTR8/SV-neo in similar assays. We found that PE serum also induced the UPR pathway in trophoblast cells, increasing the expression of target genes. Figure 1E and Supplementary Table 3 shows high gene expression levels of *GADD34*, *CHOP*, and *SDF2* after 12 h and 24 h of serum treatment. ATF4, the functional pathway for cell survival under ER stress, was also induced in this trophoblast cell line. Upregulation of *CHOP* was greater in 12 h, decreasing by 24 h, although it remained significantly different from controls (*p* = 0.003; Figure 1E).

### Sera From Pregnant Women With Preeclampsia Change the Metabolic Activity and Cell Death Rates in Chorionic Explants

**FIGURE 2:**
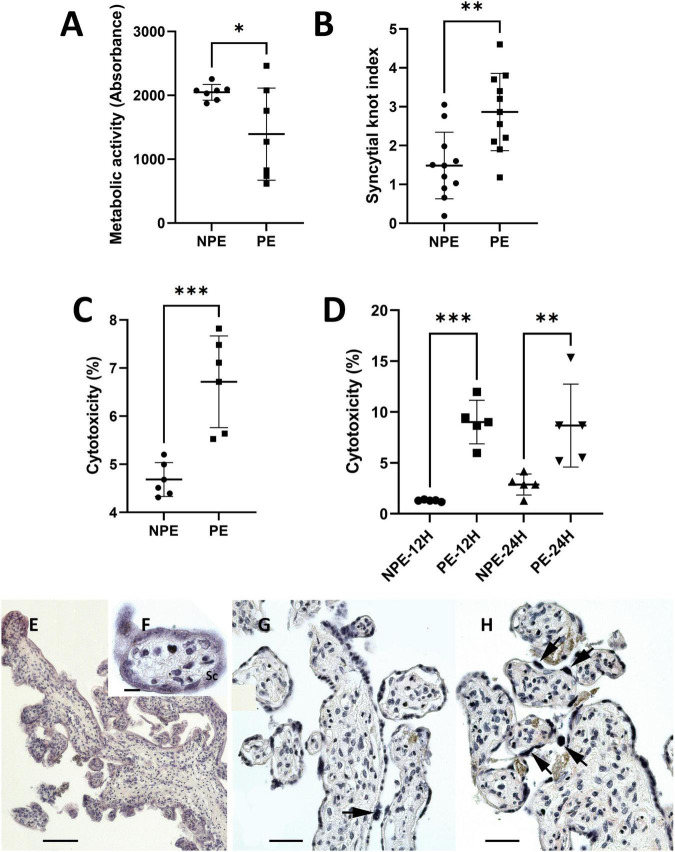
Influence of PE serum on placental explants **(A–C)** and HTR8/SV-neo trophoblast cells **(D)**. **(A)** Mitochondrial metabolic activity of placental explants treated with PE serum for 24 h. Controls were prepared with serum from NPE pregnant women. Placental PE-treated samples showed lower viability than control (*n* = 7). **(B)** Syncytial knots index in PE-serum treated and control placental explants (*n* = 11). **(C,D)** LDH levels released by placental explants **(C)**, 24 h, *n* (6) and HTR8 cells **(D)** 12 and 24 h, *n* (5) after treatment with PE serum. LDH release was higher in PE serum-treated cultures than in controls. Data were analyzed using the unpaired Student’s *t*-test followed by the Mann-Whitney posttest. The values are represented as mean ± SD. The experiments were repeated at least three times on different occasions. ^∗^*p* < 0.01; ^∗^*p* < 0.05, ^∗∗∗^*p* < 0.005. **(E–H)** H.E.-stained sections of 24 h-cultured explants with NPE serum **(E–G)** and PE serum **(H)**. The morphology of the explants appears to be relatively normal **(E–G)**. Syncytial knots (arrows) were more evident in PE-serum treated explants **(H)**. Scale bars represent 200 μm in **(E)** 20 μm in **(F)**, and 100 μm in **(G,H)**.

Placental metabolic activity and cell death were determined by MTT and LDH release assays, respectively, comparing placental explants incubated with NPE and PE serum from pregnant women. Incubation with PE serum reduced metabolic activity of the chorionic villi by around 32% (1,393 ± 718.3 vs. 2,048 ± 123.8, *p* < 0.01; *n* = 7) when compared NPE serum treatment (Figure 2A). PE serum also impaired cell viability by approximately 30,5% (6.72 ± 1.06 vs. 4.68 ± 0.32, *p* < 0.005; *n* = 6, Figure 2C). Similarly, the PE-treated trophoblast cell lineage HTR8/SV-neo (*n* = 5) also exhibited higher levels of LDH release after 12 h (9.0 ± 2.4 vs. 1.3 ± 0.1; *p* < 0.005), and 24 h of treatment (8.8 ± 4.5 vs. 2.8 ± 1.2; *n* = 5, *p* < 0.05) than NPE serum-treated cultures (Figure 2D). The quantitative morphological analysis also showed a significantly increased number of syncytial knots (*p* = 0.014, Figures 2B,E–H) in the villi treated with PE serum.

### Endoplasmic Reticulum Stress May Cause Adverse Effects on the Transcription of Genes Involved in Angiogenesis

**FIGURE 3:**
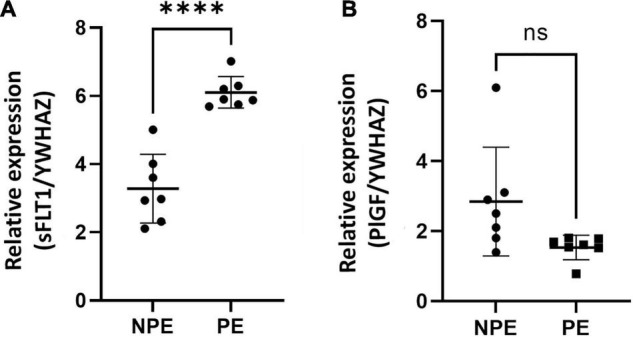
Gene expression of *sFlt1* **(A)** and *PlGF* **(B)** vasoactive factors in placental explants cultured with the serum of NPE and PE patients. Gene expression of *sFlt1* **(A)** was higher (^∗∗∗∗^*p* < 0.0001) in explants PE serum-treated in comparison with controls (NPE). Data were analyzed using the unpaired Student’s *t*-test followed by the Mann-Whitney test. The values were represented as mean ± SD; *n* = 7.

Gene expression of both PlGF and sFLT1 was found at basal levels in control chorionic villi, but it underwent significant changes with PE serum (Figure 3). Placental pro-angiogenic PlGF mRNA expression did not change when the villous were treated with PE serum. Moreover, PE serum–treated villi showed significantly increased sFLT1 gene regulation (*p* < 0.0001, *n* =7).

### Spectral Features of Preeclampsia Serum

**FIGURE 4:**
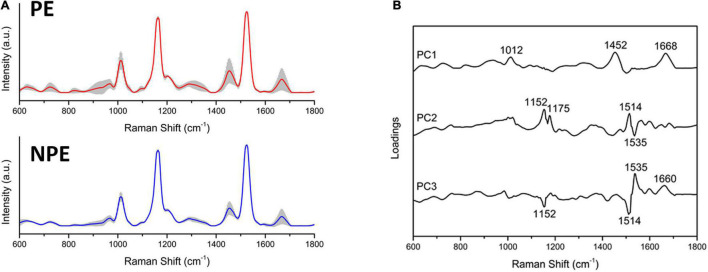
Preeclamptic and non-preeclamptic serum biochemical signatures. **(A)** The Raman spectra represent the averages of Raman spectra in the fingerprint region (600–1,800 cm^–1^). The shaded areas correspond to the Raman bands where the main variances observed in the samples occurred. **(B)** Loadings of PC1, PC2, and PC3 for NPE and PE serum.

Raman spectroscopy identified the most critical spectral differences between control and PE serum. A total of five spectra were acquired from each sample; the average Raman spectra and the most variable areas are depicted in Figure 4A and Supplementary Figure 1A. PCA was used to classify and interpret the spectral data. A three-dimensional plot was constructed with combinations of sets of scores of the first three PCs. The first three PCs explained 97% of the variance of the original data set, with PC1 describing 91%, PC2 describing 3%, and PC3 describing 3% of the total variance (Supplementary Figure 1B). Nevertheless, the PCA did not divide the two groups into two different clusters, indicating that both groups are not different among themselves (Supplementary Figure 1B). The corresponding plots of the PC1, PC2, and PC3 loadings were used to determine the differentiation capability of PCA and identification of significant Raman features (Figure 4B and Supplementary Figure 1A). The PC loadings represent the biochemical differences between the groups. From the most significant changes in PCA, the Raman bands were assigned for their main contributors (Supplementary Table 4 and Supplementary Figure 1C) based on the literature (De Gelder et al., 2007; Movasaghi et al., 2007; Basar et al., 2012; Czamara et al., 2015; Surmacki et al., 2015; Talari et al., 2015; Kelestemur and Çulha, 2017; Nguyen et al., 2017; Balan et al., 2019; Bai et al., 2020).

The preeclamptic serum had bands increased in relation to the control that assigned to the following: DNA backbone and RNA (*p* = 0.0202); tryptophan (*p* = 0.0214); α-helix from amide III (*p* < 0.0001); amide I and lipids (*p* = 0.0085); and β-sheet from amide I (*p* = 0.0085). Moreover, the PE serum reduced intensity in bands assigned to the following: tyrosine and arginine (*p* = 0.0214); tyrosine, leucine, and phenylalanine (*p* = 0.0015); and carotenoids (*p* = 0.001) (Supplementary Table 4).

## Discussion

### Characteristics of the Study Population

The clinical characteristics of the patients with PE selected for this study are in accordance with previous reports (Sarno et al., 2015; Dong et al., 2017; Brown et al., 2018). In these pregnant women, gestational age, the number of pregnancies, the number of previous deliveries, and fetal weight were lower than NPE patients, and blood pressure levels and proteinuria were higher. Primiparous women have an increased risk for preeclampsia and premature delivery (Sibai et al., 2005), which may explain this predominance in our selected PE group. Similarly, hypertension that is a hallmark of this disorder (Brown et al., 2018). Although proteinuria is no longer one of the essential criteria for the clinical diagnosis of preeclampsia, its levels are essential for predicting adverse maternal and fetal outcomes (Dong et al., 2017). According to Sarno et al. (2015), maternal and fetal health during and after pregnancy are dramatically compromised when early PE is associated with early proteinuria in addition to hypertension, both characteristics of severity we also found in our patients.

### Preeclampsia Serum Induced Gene and Protein Expression of Endoplasmic Reticulum Stress and Unfolded Protein Response Biomarkers in Placental Cells

ER stress induction by PE serum was evidenced by the expression of critical targets of ER stress signaling, as *GRP78, CHOP, GADD34*, and *SDF2* and phosphorylation of eIF2α (Xu et al., 2005; Burton and Yung, 2011; Lorenzon-Ojea et al., 2020; Lorenzon et al., 2021).

Increased expression of GRP78 was previously found in placentas from patients with severe early and late-onset PE, suggesting that in a preeclamptic environment GRP78 remains constantly activated to maintain placental homeostasis (Yoshida, 2007; Burton and Yung, 2011; Yung et al., 2014; Fu et al., 2015; Mochan et al., 2019; Lorenzon-Ojea et al., 2020).

Phosphorylation of eIF2α is a critical event in ER stress induction initiated by PERK signaling (Metcalf et al., 2020). It is mediated by the dissociation of GRP78/BiP from the PERK-sensing domain localized to the ER lumen, which leads to the activation of eIF2α through a mechanism involving specific phosphorylation of serine 51 (Metcalf et al., 2020). This phosphorylation leads to eIF2α inactivation and inhibition of ribosomal translation initiation and transient attenuation of selected new protein synthesis (Walter and Ron, 2011; Metcalf et al., 2020). The increased elF2α phosphorylation in our results therefore indicate UPR activation of the PERK branch (PERK/p-elF2α) in the villous explants PE-serum treated.

Although PERK signaling and phosphorylation of eIF2α induce a global downregulation of translation, selected mRNAs can escape, including the activation of *ATF4*. It is upregulated during ER stress, and, among other integrated stress responses, it can induce apoptosis by upregulating *CHOP* (Xu et al., 2005). Despite the interference in elF2α phosphorylation, in our assays we did not detect changes in ATF4 expression during the time-pointed selected in our experimental design. One possible explanation may be the activation of proteasomal degradation post-translationally induced by elF2α phosphorylation (Ameri and Harris, 2008). In this context, it is possible that the stimulus given by the PE serum has induced a transient increase in *ATF4* expression, returned to basal lines after 24 h of culture upon control of proteasomal degradation.

However, ER stress and UPR may diverge the signaling pathways depending on the stressor determining differences in the cellular fate. Spliced XBP1 may act on recovery of homeostasis by activation of the IRE-1 pathway, encoding a transcription factor that upregulates UPR target genes, including genes that function in the ER-associated protein degradation system to protect cells from ER stress-induced apoptosis (Yoshida, 2007). Our results demonstrated that GRP78 and elF2a responded to the PE stress inducer, whereas it seems to fail to induce ATF4 and spliced XPB1. Since both are closely related to cell survival mechanisms, it is also possible that the UPR in the villous cells downregulated these factors, shifting to apoptosis rather than cell survival when stressed by severe PE serum.

As a transcription factor, *CHOP* can negatively regulate the expression of anti-apoptotic genes in the Bcl-2 family (*BCL-2, BCL-X, BCL-W*) and sensitize cells to apoptosis when ER functions are severely impaired (McCullough et al., 2001). The increase in CHOP expression in our assays also favors this possibility. In addition, recent evidence shows the activation of the PERK branch correlated to SDF2 expression in cell lines and placental tissues in disorders such as severe preeclampsia (Lorenzon-Ojea et al., 2016; Lorenzon et al., 2021). Silencing *SDF2* gene expression gave rise to changes in UPR cell survival/apoptosis markers, characterizing this protein as a pro-apoptotic factor (Lorenzon-Ojea et al., 2016). The present study results also found an increase in SDF2 protein and gene levels after PE serum insult. Therefore, our findings suggest that at least the PERK branch of the UPR is associated with PE serum insult in the chorionic villus, where *CHOP* and *SDF2* play pivotal roles in terminal UPR transduction. Because both transcriptionally activate the expression of pro-apoptotic factors (Sano and Reed, 2013; Lorenzon-Ojea et al., 2016; Ibrahim et al., 2019), PE serum may be participating in this process.

The chorionic villus is a complex tissue in which trophoblast, endothelial cells, and macrophages play essential roles (Choudhury et al., 2017). To investigate whether the response seen with PE serum had the participation of the trophoblast cells, we use the non-malignant trophoblast cell line HTR8/SV-neo in similar assays. As well chorionic villous explants, we also observed an induction of the UPR signaling pathway at gene and protein levels in the trophoblast cells. The adaptive UPR response was seen as a late expression of *GADD34* and *ATF4*, and an unbalanced higher expression of *SDF2*, up to a fivefold increase relative to the culture’s control and early time. The expression of *CHOP* and *SDF2* in villous tissues and trophoblast cells also suggests a pro-apoptotic effect of CHOP downstream of ER stress activation (Oyadomari and Mori, 2004) occurring upon PE serum insult, but also involving the activation of ATF4, primarily associated with protein homeostasis, in the trophoblast cell line. The use of the trophoblast cell line undoubtedly showed the sensitivity of the trophoblast to respond to the serum of pregnant women with PE, adding its contribution to the UPR in the chorionic villi. These cells, however, do not undergo syncytialization to form the syncytial layer, the master component of the villous barrier. This different cellular profile may have contributed to a differential adjustment of ATF4 expression in response to PE serum.

### Sera From Pregnant Women With Preeclampsia Change the Metabolic Activity and Cell Death Rates in Chorionic Explants

Because increased CHOP and SDF2 expression after PE serum might indicate a cell death program activated through the PERK branch of the UPR pathway, we also investigated the cellular response of PE serum on mitochondrial metabolism and cellular viability. Our data show decreased metabolic activity and cell viability, which is consistent with the influence of PE serum on placental villi and may have been activated by increased CHOP expression as observed in other models (Zinszner et al., 1998; Lorenzon-Ojea et al., 2016).

Syncytial knots are nuclear aggregates of condensed chromatin within the syncytial layer that, when particularly pronounced, are associated with placental pathology (Loukeris et al., 2010). We measured the knotting index to assess severity (Norah et al., 2013). We observed they were more often in PE-treated villi than in controls, which reinforces the role of PE serum adversely affecting placental physiology.

### Endoplasmic Reticulum Stress May Cause Adverse Effects on the Transcription of Genes Involved in Angiogenesis

Disturbances in the placental expression of angiogenic factors negatively affecting maternal vessels have been considered crucial in the pathogenesis of PE (Wang et al., 2009). The unbalanced production, particularly the ratio between soluble FLT1 and PlGF, has been considered a major factor in the direct action on the maternal endothelial activation and is therefore considered valuable as a prognostic marker (Nikuei et al., 2015). The binding of sFLT1 with the cell surface of circulating VEGF and PIGF leads to a dominant-negative effect that makes endothelial cells more sensitive to low TNF levels (Zárate et al., 2014). We reported that sFlt1 gene expression in PE serum-treated samples was significantly increased relative to controls, suggesting this serum could also influence the balance of angiogenic factors in placental cells. This increase may be of paramount value. The possibility that the PE serum can act as a positive regulator of placental sFLT1 mRNA suggests that continuous and sustained activation may occur at the placental barrier, lasting and eventually aggravating the angiogenic unbalance in the severe PE. In addition, translation of mRNAs for VEGF and FGF2 responding to several physiological and pathological conditions has already been shown in cell lines and mice through ER stress activation PERK branch (Philippe et al., 2016). In this context, our result might be related to a potential action of PE serum over placental angiogenic balance resulting from UPR activation, an encouraging starting point for further research.

### Spectral Features of Preeclampsia Serum

Our results corroborate Basar et al. (2012) and Chen et al. (2012) regarding the changes in bands related to amino acids and lipids in the serum of preeclamptic patients. Nevertheless, the observation reported here also included modifications in DNA/RNA and carotenoids. Changes in band intensities of amino acids and amide were attributed to differences in protein conformation and, therefore, putative structural disorders (Chen et al., 2012). Unusual spectra for phenylalanine and tryptophan were also correlated to oxidative modifications, common in preeclampsia (Chen et al., 2012). Our data add new information consistent with PE, also showing a reduction in carotenoid structural levels. Carotenoids are usually associated with a protective effect against some chronic diseases as part of an antioxidant defense system, efficiently scavenging singlet molecular oxygen and peroxyl radicals (Stahl and Sies, 2003; Gomes, 2007). Lower maternal serum levels of β-carotene and lycopene have already been reported in preeclamptic pregnancy, associated with increased oxidative stress and a reduced antioxidant plasma levels commonly found in this disorder (Mikhail et al., 1994; Palan et al., 2001). In addition, we found changes in DNA/RNA in the serum of PE patients. There is substantial evidence that increased concentrations of maternal plasma cell-free genomic DNA and RNA in PE correlated with disease severity (Kolarova et al., 2021). Although the present study did not characterize the fetal or maternal source of DNA and RNA molecules, the fetus may have a considerable contribution. Increased plasma fetal RNA has been shown in levels tenfold higher in pregnant women with preeclampsia (Hahn et al., 2011), many of which belong to the microRNA family (Mayor-Lynn et al., 2011). The functional significance of the spectral modifications in these molecules remains to be elucidated. The Raman spectrum provides an empirical signature of three-dimensional molecular structure and dynamics, in which conformational changes can disable or activate essential binding domains (and consequently functional activities). In this study, we showed structural changes in the spectra of amino acids, carotene, lipids, and DNA/RNA in the serum of pregnant women with PE. Some of these modifications may be associated with the induction of ER stress found in chorionic villi treated with this serum. However, while a direct implication is tempting, further studies are needed to determine whether these altered candidates can specify outcomes in the progression and severity of PE.

## Conclusion

**FIGURE 5:**
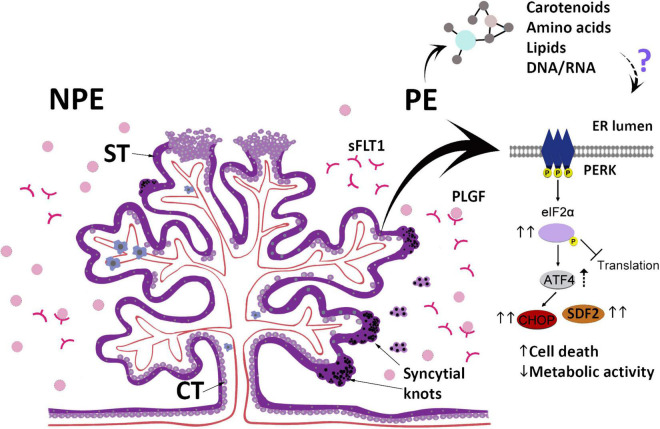
The scheme summarizes the main findings in this study and highlights the changes in the UPR pathway after culturing placental explants (chorionic villous) obtained from non-preeclamptic (NPE) pregnant women with preeclamptic serum (PE). ST, syncytiotrophoblast; CT, villous cytotrophoblast; sFLT1, Soluble fms-like tyrosine kinase-1; PlGF, Placental growth factor.

This study provides evidence that the serum of pregnant women with PE may impact placental villi, changing morphology, viability, and secreted functional factors while triggering ER stress and a response to the unfolded proteins (Figure 5). The differences between PE and control sera include molecules that may act as inducing factors in these processes. In summary, the results obtained in our assays suggest that after the development of PE, the serum profile of pregnant women may be an additional factor that feeds a continuous imbalance of placental homeostasis. In addition, this study may expand the possibilities for understanding the pathogenesis of this disorder.

## Data Availability Statement

The original contributions presented in the study are included in the article/Supplementary Material, further inquiries can be directed to the corresponding author/s.

## Ethics Statement

The studies involving human participants were reviewed and approved by the Brazilian National Ethics Committee of Human Experimentation. The patients/participants provided their written informed consent to participate in this study.

## Author Contributions

EB conceived the study. KC and KP carried out the experimental assays and its analysis. AB, AM, EF, and ES carried out the RAMAN spectroscopy assays and data analysis. RF, MZ, MH, and EA selected the patients. KP, AL, and EB participated in study design, and coordinated and drafted the manuscript. All authors have revised and approved the final version of the manuscript.

## Conflict of Interest

The authors declare that the research was conducted in the absence of any commercial or financial relationships that could be construed as a potential conflict of interest.

## Publisher’s Note

All claims expressed in this article are solely those of the authors and do not necessarily represent those of their affiliated organizations, or those of the publisher, the editors and the reviewers. Any product that may be evaluated in this article, or claim that may be made by its manufacturer, is not guaranteed or endorsed by the publisher.

## Supplementary Material

Supplementary Figure 1 Raman spectral changes in preeclamptic serum. **(A)** Main Raman spectra from control (NPE) and preeclamptic (PE) serum with the most variable bands. **(B)** Three-dimensional PCA score plot. **(C)** Raman intensity from the bands: 629, 724, 966, 1,152, 1,175, 1,201, 1,452, 1,514, 1,525, 1,535, 1,660, and 1,668 cm^–1^. Bar graphs represent mean values ± S.E.M. ^∗^*p* < 0.05, ^∗∗^*p* < 0.01, ^∗∗∗^*p* < 0.001.

## Abbreviations

ATF4: activating transcription factor 4
ATF6: activating transcription factor 6
BiP/GRP78: binding immunoglobulin protein/78 kDa glucose-regulated protein
CHOP: C/EBP-homologous protein
eIF2α: eukaryotic translation initiator factor 2α
ER: endoplasmic reticulum
ERAD: ER-associated protein degradation
IRE1: inositol-requiring enzyme 1
PERK: protein kinase R (PKR)-like ER kinase
UPR: unfolded protein response
XBP1: X-box binding protein
PlGF: placental growth factor
sFlt1: soluble fms-like tyrosine kinase-1
GADD34: growth arrest and DNA damage-inducible 34
SDF2: stromal-derived factor 2
UPR: unfolded protein response
BCL2: B-cell lymphoma-2.
