# Supplementary figures and images for: Serum From Preeclamptic Women Triggers Endoplasmic Reticulum Stress Pathway and Expression of Angiogenic Factors in Trophoblast Cells
Source: Front Physiol. 2022 Feb 4;12:799653. doi: 10.3389/fphys.2021.799653 (PMC8855099; doi:10.3389/fphys.2021.799653)

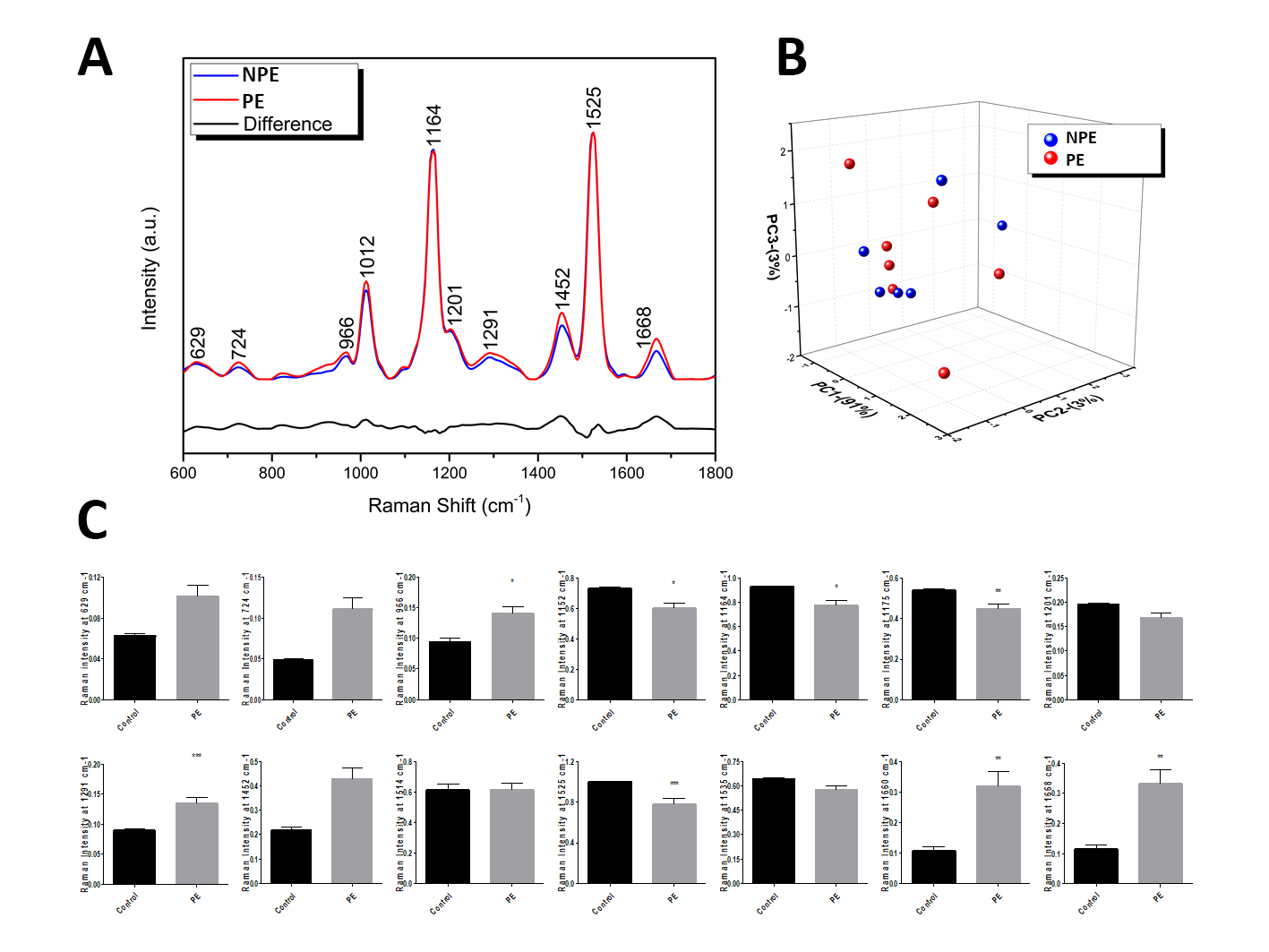

Supplement: Supplementary Figure 1 — Raman spectral changes in preeclamptic serum. (A) Main Raman spectra from control (NPE) and preeclamptic (PE) serum with the most variable bands. (B) Three-dimensional PCA score plot. (C) Raman intensity from the bands: 629, 724, 966, 1,152, 1,175, 1,201, 1,452, 1,514, 1,525, 1,535, 1,660, and 1,668 cm–1. Bar graphs represent mean values ± S.E.M. ∗p < 0.05, ∗∗p < 0.01, ∗∗∗p < 0.001. [file Image_1.TIF]
