# Supplementary material for: Serum From Preeclamptic Women Triggers Endoplasmic Reticulum Stress Pathway and Expression of Angiogenic Factors in Trophoblast Cells
Source: Front Physiol. 2022 Feb 4;12:799653. doi: 10.3389/fphys.2021.799653 (PMC8855099; doi:10.3389/fphys.2021.799653)
Supplement: Supplementary file 2 [file Data_Sheet_1.PDF]

Supplementary Table S1 –Sequences of gene-specific primers used for quantitative real-time PCR analysis.

Target	Sequences (5' - 3')	Annealing temperature
SDF2	F: 5' - AGCTTCAGCCTGCACATTTGAAC - 3'<br>R: 5' - TCCATGATGCCAAGCTCCTGAAGA - 3'	60 ∘C
sXBP1	F: 5' - TGCTGAGTCCGCAGCAGGTG - 3'<br>R: 5' - GCTGGCAGGCTCTGGGGAAG - 3'	62 ∘C
CHOP	F: 5' - TTAAGTCTAAGGCACTGAGCGTATC - 3'<br>R: 5' - TGCTTTCAGGTGTGGTGATG - 3'	56 ∘C
GADD34	F: 5' - AGGACACAGAGGAAGAGGAAG - 3'<br>R: 5' - TGTAGCAGGAGTGGAAGAGG - 3'	60 ∘C
ATF4	F: 5' - GACGGAGCGCTTTCCTCTT - 3'<br>R: 5' - TCCACAAAATGGACGCTCAC - 3'	58 ∘C
PlGF	F: 5'- GTCACCATGCAGCTCCTAAA - 3'<br>R: 5'- CCTTTCCGGCTTCATCTTCT - 3'	60 ∘C
sFlt-1	F: 5'- AGAGGTGAGCACTGCAACAA - 3'<br>R: 5' - GTGGTACAATCATTCCTTGTGCT - 3'	60 ∘C
YWHAZ	F: 5' - GCCACAATGTTCTTGGCCCATCAT - 3'<br>R: 5' - TGGTTGGTGACAAGACAGAAGGCT - 3'	60 ∘C
