# Supplementary material for: Serum From Preeclamptic Women Triggers Endoplasmic Reticulum Stress Pathway and Expression of Angiogenic Factors in Trophoblast Cells
Source: Front Physiol. 2022 Feb 4;12:799653. doi: 10.3389/fphys.2021.799653 (PMC8855099; doi:10.3389/fphys.2021.799653)
Supplement: Supplementary file 3 [file Data_Sheet_2.PDF]

Supplementary Table S2 –Primary and secondary antibodies used for western blot analysis.

Target	Species	Manufacturer	Dilution
GRP78	Goat anti-human	(sc-1050) Santa Cruz, Dallas, Texas, USA	1:1000
eIF2α	Rabbit anti-human	(#9722) Cell Signalling Technology, Danvers, USA	1:1000
p-eIF2α	Rabbit anti-human	(#9721) Cell Signalling Technology, Danvers, USA	1:500
SDF2	Rabbit anti-human	(AV48718) Sigma-Aldrich, Saint Louis, MO, USA	1:1000
β-Actin	Mouse anti-human	(ab8227) Abcam, Cambridge, UK	1:5000
anti-goat IgG-HRP	Rabbit	(AP106P) Sigma-Aldrich, Saint Louis, MO, USA	1:1000
anti-rabbit IgG-HRP	Goat	(12-348) Sigma-Aldrich, Saint Louis, MO, USA	1:1000
anti-mouse IgG-HRP	Goat	(074-1806) KPL, Milford, MA, USA	1:1000
