# Supplementary material for: Serum From Preeclamptic Women Triggers Endoplasmic Reticulum Stress Pathway and Expression of Angiogenic Factors in Trophoblast Cells
Source: Front Physiol. 2022 Feb 4;12:799653. doi: 10.3389/fphys.2021.799653 (PMC8855099; doi:10.3389/fphys.2021.799653)
Supplement: Supplementary file 4 [file Data_Sheet_3.PDF]

Supplementary Table S3. Real-time quantitative RT-PCR and western blot analysis raw data to determine the level of ER stress and UPR target genes and proteins in control and PE-treated placental explants and HTR8/SV-neo cells.

**Relative gene expression**						
	**Villous (n=7)**		**HTR8/SV-neo (n=5)**			
	**Ct-24h**	**Treated-24h**	**Ct-12h**	**Treated-12h**	**Ct-24h**	**Treated-24h**
GADD34	1.04 ± 0.17	2.57 ± 0.57[a]***	1.02 ± 0.23	4.70 ± 0.55[a]***	1.05 ± 0.30	11.60 ± 2.91[a, b]***
CHOP	0.96 ± 0.29	1.89 ± 0.34[a]**	1.98 ± 1.18	10.57 ± 5.03[a]***	1.16 ± 0.70	6.79 ± 2.62[a]**
SDF2	1.02 ± 0.22	1.63 ± 0.40[a]*	0.73 ± 0.20	0.54 ± 0.06	1.02 ± 0.20	4.01 ± 1.38[a]***, [b]***
sXBP1	1.03 ± 0.28	0.80 ± 0.33	-	-	-	-
ATF4	0.84 ± 0.32	1.09 ± 0.10	1.46 ± 1.27	3.50 ± 0.63*	1.01±0.17	5.35 ± 1.18[a]***, [b]*
**Relative protein expression**						
SDF2	0.37 ± 0.1	1.05 ± 0.5***	0.80 ± 0.11	0.98 ± 0.11	0.81 ± 0.23	1.30 ± 0.06[a]***, [b]*
GRP78	1.00 ± 0.38	1.67 ± 0.55*				
eIF2α	0.63 ± 0.19	0.52 ± 0.12	0.70 ± 0.08	0.69 ± 0.17[a]*	0.93 ± 0.17	0.81 ± 0.14
p-eIF2α	0.37 ± 0.16	0.64 ± 0.17**	0.65 ± 0.18	1.06 ± 0.24[a]*	0.76 ± 0.19	0.58 ± 0.17[b]**

Data are means ± SD (n=7). Data from villous cultures were analyzed using Student's unpaired *t*-test followed by Mann-Whitney posttest to compare treated (PE serum) and control (serum from NPE pregnancy). Time points of the HTR8/SV-neo cultures were analyzed using ANOVA and Tukey's multiple comparisons post hoc test. a, compared to respective control (NPE); b, compared to 12h of treatment with PE serum. *$p<0.05$; **$p<0.005$; ***$p< 0.0005$.
