# Supplementary material for: Serum From Preeclamptic Women Triggers Endoplasmic Reticulum Stress Pathway and Expression of Angiogenic Factors in Trophoblast Cells
Source: Front Physiol. 2022 Feb 4;12:799653. doi: 10.3389/fphys.2021.799653 (PMC8855099; doi:10.3389/fphys.2021.799653)
Supplement: Supplementary file 5 [file Data_Sheet_4.PDF]

Supplementary Table S4: Raw data of Raman peaks positions and their respective assignments and contributions

**Raman band position [cm^{-1}]**	**Band assignment**	**Contributions**	**Control bands intensities** (mean ± SEM)	**Preeclamptic bands intensities** (mean ± SEM)	**P**
**629**	β(C-C)	Uric acid	0.0622 ± 0.0029	0.1013 ± 0.0109	0.0525
**724**	βCH	Acetyl-CoA, CoA, Methionine, Sphingomyelin	0.0486 ± 0.0021	0.1108 ± 0.0139	0.0514
**966**	υ(C-C)	DNA backbone, RNA	0.0944 ± 0.0056	0.1406± 0.0112	0.0202
**1012**	Strong Van der Walls force interactions	Tryptophan	0.3606 ± 0.0096	0.4458 ± 0.0200	0.0214
**1152**	υ(C-C)	Carotenoids	0.7305 ± 0.0066	0.5993 ± 0.0331	0.0298
**1164**	Ring breathing modes	Tyrosine and Arginine	0.9305 ± 0.0035	0.7742 ± 0.0412	0.0214
**1175**	βCH	Tyrosine, Leucine, Phenylalanine	0.5388 ± 0.0062	0.4480 ± 0.0212	0.0015
**1201**	υCH	Tyrosine	0.1953 ± 0.0026	0.1672 ± 0.0100	0.9788
**1291**	βCH	Amide III (α-helix)	0.0893± 0.0035	0.1347 ± 0.0091	< 0.0001
**1452**	β$(CH)_2$	Valine	0.2183 ± 0.0135	0.4273 ± 0.0460	0.1327
**1514**	υ(C=C)	Carotenoids	0.6120 ± 0.0402	0.6153 ± 0.0394	0.9683
**1525**	υ(C=C)	Carotenoids	0.5099 ± 0.0277	0.3385 ± 0.0272	0.001
**1535**	υ(C=C)	Carotenoids (β-carotene)	0.6428 ± 0.0060	0.5765 ± 0.0234	0.4537
**1660**	υ(C=C)	Amide I, Lipids	0.1057 ± 0.0146	0.3185 ± 0.0473	0.0085
**1668**	υ(C=C)	Amide I (β-sheet)	0.1145 ± 0.0155	0.3311 ± 0.0048	0.0085

β-bending; υ-stretching; $υ_{a-}$ asymmetric stretching (Bai *et al.* 2020; Balan *et al.* 2019; Başar *et al.* 2012; Czamara *et al.* 2015; Gelder *et al.* 2007; Keleştemur *et al.* 2018; Movasaghi *et al.* 2007; Nguyen *et al.* 2017; Surmacki *et al.* 2015; Talari *et al.* 2014). $p < 0.05$ in two-tailed Mann-Whitney test.
